# Antenatal iron and folic acid supplementation use by pregnant women in Khartoum, Sudan

**DOI:** 10.1186/1756-0500-7-498

**Published:** 2014-08-07

**Authors:** Hala Abdullahi, Gasim I Gasim, Ahmed Saeed, Abdulmutalab M Imam, Ishag Adam

**Affiliations:** 1 Department of Obstetrics and Gynecology, Faculty of Medicine, University of Khartoum, Khartoum, Sudan; 2 Medical College, Qassim University, Gassim, Saudi Arabia

**Keywords:** Folic acid, Iron, Pregnant women, Sudan

## Abstract

**Background:**

Anaemia during pregnancy can lead to adverse maternal and perinatal outcomes. The WHO recommends that all pregnant women in areas where anaemia is prevalent receive supplements of iron and folic acid. However, due to many factors, the use of iron and folic acid supplementation is still low in many countries. This study was conducted to assess the rates of iron-folic supplementation and the associated factors during pregnancy and the effects of taking iron-folic acid supplementation on rates of maternal anaemia and low birth weight (LBW) infants.

**Methods:**

A cross-sectional study was conducted at Khartoum Hospital, Sudan. Enrolled women answered a questionnaire on socio-demographics characteristics, their pregnancy and delivery.

**Results:**

Of 856 women, 788 (92.1%) used iron-folic acid supplementation during pregnancy and 65.4% used folic acid. While place of residence, occupation and level of education were not associated with iron-folic acid usage, older age (OR = 3, CI = 1.4–6.3) and use of antenatal care (OR = 14.3, CI = 7.4–27.5) were associated with iron-folic acid use. Primiparity (OR = 3.8, CI = 1.9–7.6), maternal employment (OR = 3.9, CI = 2.25–6.77) and use of antenatal care (OR = 7.9, CI = 4.1–15) were the factors associated with folic acid. Using iron-folic acid was protective against anaemia (OR = 0.39, CI = 0.2–0.7) and LBW infants (OR = 0.3, CI = 0.17–0.68).

**Conclusion:**

There was a high rate of iron-folic acid supplementation use among pregnant women in Khartoum, Sudan, which was beneficial in preventing anaemia in expectant mothers and infants of LBW.

## Background

The World Health Organization (WHO) estimates that 500 million women of reproductive age worldwide are anaemic, and the vast majority of anaemic pregnant women are in Africa [1,2]. Anaemia during pregnancy (haemoglobin level less than 11 g/dl) can lead to adverse maternal and perinatal outcomes, including maternal and perinatal mortality [3-8].

The WHO recommends that all pregnant women in areas where anaemia is prevalent should receive supplements of iron and folic acid [5]. In spite of the WHO recommendation, the use of iron and folic acid supplementation is still low in many countries, especially in countries with low resources [9,10]. Various factors, such as socio-demographics and health factors, determine the compliance and adherence of pregnant women to the iron and folic acid supplementation [11-16].

Anaemia during pregnancy is a major health problem in Sudan affecting around half of women in some areas; it is a risk factor for maternal and perinatal morbidity and mortality [3,8]. Pregnant women are at risk of anaemia regardless of their age or parity [8,17]. However, no published data exist on the use of iron-folic acid supplements in pregnant women in Sudan. Such studies are needed to generate data for health planners and program managers.

Thus, this study was conducted to investigate the rates and predictors of iron-folic supplementation during pregnancy and the effect of the supplement on the prevalence of anaemia in pregnant women and the incidence of low birth weight (LBW) infants.

## Methods

This hospital-based cross-sectional study was carried out between June and August 2012 at Khartoum Hospital, Sudan. The hospital is one of 3 central hospitals in Khartoum province serving the local community and receiving medical referrals from other parts of Sudan.

Women carrying a singleton pregnancy were enrolled in the study. Each participant provided written, informed consent. A medical officer used a pre-tested questionnaire to gather data from each parturient mother on her age, parity, level of education (illiterate, primary school or secondary school education and above), antenatal care visits, occupation (house wife or working mother), gestational age in weeks, medical diseases (diabetes, hypertension, urinary tract infections and thyroid problems) use of iron-folic acid supplements, delivery data including the sex and the birth weight of the baby. The maternal haemoglobin level, which is routinely checked for all women admitted in labor using automated hematology analyzer (Sysmex KX21N: Sysmex Corporation, Kobe, Japan), was recorded.

The term ‘supplement use’ refers to the use of iron-folic acid or folic acid only. Combined tablets containing both supplements are available free of charge as part of a antenatal care package, which is also free of charge. Governmental policy recommends the use of folic acid in early pregnancy (the first trimester) and the combined tablets that contain iron and folic acid thereafter (in the second and third trimesters.

A birth weight of less than 2500 g is used as a cut off limit to define low birth weight (LBW) in this study.

The sample size of 850 women was calculated to have an 80% power to detect a difference of 5% (5% at α = 0.05) in the proportion of those using the supplement and those who did not, assuming the true proportion was 70% and that 10% of women would not be respond.

Data were analyzed using SPSS version 20.0 for Windows (SPSS Inc., Chicago, IL, USA). Mean (SD) and proportions of the variables were compared using student t-tests and χ^2^ tests, respectively. The number of women who used folic acid, or iron or both supplements during the antenatal period was calculated. Univariate and multivariate analyses were performed where iron-folic acid supplementation was the dependent variable and socio-demographic factors were the independent variables. All the independent variables in the univariate analysis were entered in the multivariate one regardless to their significant. The corrected odds ratio for anaemia and LBW were analyzed for supplements using logistic regression models. A *P*-value <0.05 was considered significant.

Ethical approval for the study was obtained from Khartoum Teaching Hospital and Sudan Medical Specialization Board.

## Results

During the study, 876 eligible women attended the hospital, of which 856 provided complete data and were included in the final analyses. Of these 856 women, 788 (92.1%) used iron-folic acid during the current pregnancy, and 65.4% used folic acid in the first trimester. A significantly higher number of women who used iron-folic acid were educated, were resident in a town and had attended antenatal care, Table 1. Of these 856 women who delivered in the hospital, 791 (92.4%) attended antenatal care and 65 (7.6%) did not attend antenatal care. The supplement rate was 99.6% (788/791) and 57.0% (37/65), P < 0.001 among the women who attended antenatal care and women who had no antenatal care and delivered in the hospital, respectively. The women who used iron-folic acid supplements were significantly older than those who did not (mean age [SD] 28.3 [6.5] versus 26.4 [7.0] years; *P* = 0.022).

**Table 1:** Comparing number (%) of the maternal and infant characteristics of the group who used iron-folic supplementation to those who didn’t at Khartoum Hospital, Sudan

**Variable**	**Used iron-folic acid**	**Not used iron -folic acid**	**P**
	**(*n*** **= 788)**	**(*n *** **= 68)**	
Primiparity	97 (12.4)	7 (10.3)	0.674
Education ≥ secondary level	601 (76.3)	43 (63.2)	0.018
Maternal job (employee)	155 (19.7)	10 (14.7)	0.377
Attended antenatal care	751 (95.3)	40 (58.8)	<0.001
Urban residence	504 (64.0)	33 (48.5)	0.014
No medical diseases	732 (92.9)	65 (95.6)	0.410
Anaemia	365 (46.3)	44 (64.7)	0.001
Male gender	424 (53.8)	35 (51.5)	0.800
Low birth weight	90 (13.1)	20 (29.4)	<0.001

In the logistic regression analysis, while there was no association between parity, education level, medical diseases (included diabetes, hypertension, thyroid problems and urinary tract infections), place of residence (urban or country) or occupation and use of iron-folic acid supplementation, older age (OR = 3, 1.4–6.3) and use of antenatal care (14.3, 7.4–27.5) were associated with iron-folic acid use, Table 2.

**Table 2:** Univariate and multivariate analyses for predictors of hematinic use among pregnant women at Khartoum, Sudan

	**Univariate analysis**			**Multivariate analysis**		
**The variables**	**OR**	**95% CI**	**P**	**O R**	**95% CI**	**P**
Age > 28.0 year	2.9	1.5-5.3	0.001	3.0	1.4-6.3	0.005
primiparae	1.2	0.5-2.7	0.673	1.2	0.45-3.1	0.72
Education ≥ secondary level	1.9	1.1-3.1	0.019	0.92	1.5-4.5	0.73
Urban residence	1.84	1.12-3.0	0.016	1.0	0.55-2.1	0.86
Maternal job (employee)	0.7	0.4-1.5	0. 384	0.81	0.35-1.91	0.63
Attended antenatal care	14.7	8.1-26.4	<0.001	14.3	7.4-27.5	<0.001
No medical diseases	0.6	0.19-2.6	0.421	0.67	0.18-2.5	0.55

Similarly, in further logistic regression analysis, age, level of education, place of residence and medical diseases were not associated with folic acid use in the first trimester; whereas parity, attending antenatal care and employment were associated with folic acid use, Table 3.

**Table 3:** Univariate and multivariate analyses for predictors of folic acid use in the first trimester among pregnant women at Khartoum, Sudan

	**Univariate analysis**			**Multivariate analysis**		
**The variables**	**OR**	**95% CI**	**P**	**O R**	**95% CI**	**P**
Age > 28.0 year	1.35	0.86-2.14	0.187	1.5	0.93-2.7	0.084
primiparae	3.4	1.9-5.9	<0.001	3.8	1.9-7.6	<0.001
Education ≥ secondary level	1.04	0.75-1.45	0.7756	0.67	0.44-1	0.068
Urban residence	0.84	0.66-1.19	0.433	1.1	0.77-1.6	0.546
Maternal job (employee)	2.37	1.56-3.6	<0.001	3.9	2.25-6.77	<0.001
antenatal care	5.7	3.2-10.2	<0.001	7.9	4.1-15	<0.001
No medical diseases	1.01	0.57-1.76	0.974	1.31	0.67-2.58	0.426

Haemoglobin readings (mean [SD] 11.0 [1.1] versus 10.2 [1.4] g/dl; *P* < 0.001) and birth weight (mean [SD] 3055.8 [545.7] versus 2818.2 [647.1] g, *P* = 0.001] were significantly higher in the women who used iron-folic acid supplements than in those who did not. Likewise the prevalence of anaemia and LBW infants was higher among women who did not use the supplement, Table 1.

The women who used iron and folic acid were less likely to have anaemia (OR = 0.39, CI = 0.2–0.7, *P* = 0.004) or a baby with LBW (OR = 0.3, CI = 0.17–0.68, *P* = 0.002).

## Discussion

The main findings of the study were that 92.1% of the investigated women used iron-folic acid supplements during pregnancy and 65.4% used folic acid only in the first trimester of their pregnancy. Older age and use of antenatal care were associated with iron-folic acid supplementation use. Those women who used iron-folic acid were at lower risk for anaemia and infants with LBW. The rate of using iron-folic acid in this study (92.1%) was higher than the rate reported among pregnant women in Eastern Sudan (81.5%) [17] or other African countries e.g. Nigeria, Tanzania and Kenya [9,10,18]. The high rate of iron and folic acid use in the current study compared with other studies may be because this study was carried out in a tertiary hospital in the capital of Sudan, Khartoum. Therefore, these results are not necessarily representative of practice in the community or in rural areas. In addition, this study found that women who have attended antenatal care were more likely to use iron-folic acid supplements or folic acid than those who had not attended antenatal care. Thus, successful uptake of iron-folic acid supplementation is linked to the use of antenatal care during pregnancy [19].

In the current study, in addition to the use of antenatal care, older women were 3 times as likely to use iron-folic acid supplementation as younger women and primipara were 4 times more likely than multipara to use folic acid in the first trimester. Previous studies showed that older age, parity and greater levels of education were associated with taking supplements during pregnancy [10,14]. However, factors associated with the use of these supplements may differ depending on setting.

The current study showed that haemoglobin level and infant birth weight was significantly higher in women who used iron-folic acid. Intake of iron and folic acid was associated with a lower risk of anaemia and infant LBW. Interestingly a recent study reported that the use of folic acid only was not related to LBW of the infant, and iron supplementation was associated with a lower risk of infant LBW, once other factors such as body mass index, obstetric diseases during pregnancy, weight gain during pregnancy and previous LBW were adjusted for [20]. In China, Lui *et al.* have found that iron-folic acid supplements reduced the incidence of anaemia but did not influence perinatal mortality or infant outcome [21]. However, the WHO recommendation for areas where anaemia is prevalent is that all pregnant women receive supplements of iron-folic acid in order to prevent anaemia and its maternal and perinatal complications [5].

## Conclusion

There was a high rate of iron-folic acid supplementation use among pregnant women in Khartoum, Sudan, which was beneficial in preventing anaemia in expectant mothers and infants of LBW.

## Competing interests

The authors declare that they have no competing interests.

## Authors’ contributions

HA and IA coordinated and carried out the study, and participated in the statistical analysis and procedures. GIG, AS and AMI participated in the clinical work and statistical analysis. All the authors have read and approved the final version of this manuscript.
